# Effect of Semaglutide versus placebo on prediabetes, psychotic symptoms, and quality of life (HISTORI): A randomized clinical trial among patients with schizophrenia

**DOI:** 10.1192/j.eurpsy.2025.2278

**Published:** 2025-08-26

**Authors:** N. Uhrenholt, A. Ganeshalingam, S. Arnfred, P. Gæde, J. Frystyk, N. Bilenberg

**Affiliations:** 1 Research Unit for Clinical Psychopharmacology, Psychiatry West, Region Zealand, Slagelse; 2 Child and Adolescent Psychiatry, Mental Health Services in the Region of Southern Denmark; 3 Endocrine Research Unit, Department of Endocrinology; 4 Steno Diabetes Center Odense, Odense University Hospital, Odense; 5 Psychiatric Research Unit, Copenhagen University Hospital, Psychiatry Region Zealand, Slagelse; 6 Department of Clinical Medicine, University of Copenhagen, Copenhagen; 7 Department of Cardiology and Endocrinology, Næstved, Slagelse & Ringsted Hospitals, Slagelse; 8 Department of Regional Health Research; 9 Department of Clinical Research, University of Southern Denmark, Odense, Denmark

## Abstract

**Introduction:**

Individuals diagnosed with schizophrenia experience a 2- to 3-fold higher mortality rate compared to the general population, with cardiovascular disease being the primary cause. On average, they lose about 15 years of potential life. Additionally, up to 15% of individuals with schizophrenia develop type 2 diabetes, further exacerbating their health challenges.

**Objectives:**

This study aimed to evaluate the efficacy of Semaglutide, a glucagon-like peptide-1 receptor agonist, as an adjunctive treatment to antipsychotic therapy in patients with schizophrenia spectrum disorders, prediabetes and overweight.

**Methods:**

We conducted an investigator-initiated, randomized, placebo-controlled, double-blind trial across two clinical sites in Denmark. Out of 402 possible eligible participants, 154 were enrolled. Participants were receiving second-generation antipsychotic treatment, were overweight or obese, and had prediabetes (HbA1c 39-47 mmol/mol). Data collection spanned from January 1, 2022, to May 1, 2024. The primary outcome measure was changes in HbA1c, with secondary outcomes including psychotic symptoms, quality of life, BMI, and cardiometabolic parameters. A pre-specified statistical analysis plan was registered with ClinicalTrials.gov prior to unblinding the treatment arms.

The trial is spear-headed by Odense University Hospital with recruitment from community psychiatry settings in the Region of Southern Denmark and Region Zealand.

**Results:**

The last patient visit was May 1, 2024 and unblinding occurred primo September 2024. Results will be analyzed in Q4, 2024 and primary and secondary results presented at the conference.

**Conclusions:**

Semaglutide holds potential as a novel therapeutic option for individuals with schizophrenia who experience prediabetes and antipsychotic-induced weight gain.

**Disclosure of Interest:**

None Declared

